# The Cultural Evolution of Human Nature

**DOI:** 10.1007/s10441-019-09367-7

**Published:** 2019-09-28

**Authors:** Mark Stanford

**Affiliations:** Institute of Cognitive and Evolutionary Anthropology, University of Oxford, Oxford, UK

**Keywords:** Cultural evolution, Evolutionary psychology, Cognitive anthropology, Human origins

## Abstract

Recent years have seen the growing promise of cultural evolutionary theory as a new approach to bringing human behaviour fully within the broader evolutionary synthesis. This review of two recent seminal works on this topic argues that cultural evolution now holds the potential to bring together fields as disparate as neuroscience and social anthropology within a unified explanatory and ontological framework.

**Essay review of Cecilia Heyes:*Cognitive Gadgets: The Cultural Evolution of Thinking*, Harvard University Press, 2018, 304 pp; and Robert Boyd:*A Different Kind of Animal: How Culture Transformed Our Species*, Princeton University Press, 2018, 248 pp.**

## Introduction

What makes humans different from other animals? It is an old question, and one which many have given up as meaningless or unanswerable. The simple fact of our unparalleled range and adaptability as a species cannot be denied, and indeed we seem to feel we are special for many more reasons than that. One response to this instinct is to resist it: having evolved like all other animals, our weird and wonderful panoply of behaviours is the result of neither individual creativity nor cultural supervenience, but is instead the simple product of millions of years of evolution, like the mating dance of birds or the territorial displays of octopi. If they seem unintelligible or even maladaptive, that is simply because evolution is very slow; our minds are adapted not to our present environment, but to the long vanished world of our ancestors (Cosmides and Tooby 2003). It may seem to us that behaviours such as joining religious cults, suicide bombing and racial prejudice are as irrational as they are undesirable, but in fact, on the account offered by standard evolutionary psychology, they are perfectly intelligible as the results of our ‘stone age minds’ misfiring and triggering behaviours that were adaptive long ago (Cosmides and Tooby 1987).

But this account has never sat entirely well with the vast cultural variation of our species, and in recent years, culture itself has come under a novel evolutionary lens (Henrich 2017; Richerson and Boyd 2005). Based on the insight that the class of mathematical models specified by evolutionary theory can be applied not only to genes, but to many other forms of information transmission, cultural evolutionary theory posits that human cultures do not vary, change and remain stable at random, but that cultural information is itself subject to evolutionary forces. Practices and ideas are differentially imitated and transmitted depending on unconscious cues such as the status or success of the individuals who bear them, and at times based on the way they interact with aspects of our cognitive apparatus. Cultural evolution proceeds much more quickly than genetic evolution, because individuals can change behaviours within one lifetime, and there is no need to wait for death or reproduction for the distribution of cultural traits to change. What is more, genetic and cultural evolution can interact; cultural adaptation can remove the pressure for genetic adaptation, and even change the adaptive environment to which genetic change responds.

## The Cultural Evolutionary Synthesis

In *A Different Kind of Animal* (2018), Robert Boyd, one of the founding fathers of the discipline of cultural evolution, sketches the current state of the field, and outlines his current theoretical position. Boyd places particular emphasis on blind imitation, as opposed to intelligent design, as an explanation for the diffusion of innovations throughout a population. That is, he shows that we become better adapted to our environments not by thinking consciously about how to solve problems better, but simply through evolutionary processes triggered by blind imitation.

Much evolutionary research in recent decades has been dedicated to attempting to explain co-operation, and until recently, many have argued that human co-operation is principally to be explained by reference to the small size and high degree of relatedness of hunter-gatherer groups. Large-scale co-operation may then be a result of a ‘misfiring’ of mechanisms developed to cope with that ancient environment. But it has become increasingly clear many human hunter-gatherer groups do not fit this description, and that even in the case of smaller-scale co-operation problems, many hunter-gatherer bands appear to organise co-operation through mechanisms other than kin helping and direct reciprocity (Henrich 2018). Indeed, as Boyd shows, contra the common intuition, large scale co-operation with non-kin has been extraordinarily common throughout our history as a species. Far from contributions to the public good being a ‘misfiring’ caused by evolutionary environments in which no one was a stranger, this sort of co-operation has always been with us.

The implications for evolutionary theory are profound. Boyd argues that the bulk of human co-operation is in fact undergirded not by kin helping or direct reciprocity, but by social norms: humans are extraordinarily well-adapted rule enforcers, often intrinsically motivated not only to enforce rules themselves, but also to punish others who do not punish violators. Boyd argues that the so-called ‘second-order free-rider problem’ is not a concern here; so long as only some fraction of individuals fail to punish those who do not punish, while others do so, the proportion of non-punishers should decrease with each successive iteration of the problem. Thus our strong tendency to follow and enforce social norms turns them into a powerful tool for enforcing co-operation at a large scale.

Why, then, are many norms detrimental to individuals and even maladaptive from the point of view of the individual or the group? For Boyd, the explanation lies in our exquisite ability to enforce and stabilise any norm at all; like our tendency to imitate successful others, it is content-neutral. And while our norm psychology has evolved genetically because it is highly advantageous, its content neutrality means that we are prone at times to adopt norms which themselves may not be advantageous, and may even be detrimental. The selection of adaptive versus maladaptive norms is a distinct process that takes place, Boyd argues, through cultural group selection. Those groups that adopt advantageous norms will be more likely to survive, to attract new members, and to be imitated. Thus at any given time, all manner of norms may be present across multiple groups, but cultural evolution suggests there is an ongoing process of selection and adaptation.

Neither must it be the case that maladaptive behaviours were adaptive at some point in the past. A large part of evolutionary psychology has been predicated on the assertion that much of our present-day behaviour is to be explained by reference to adaptive fitness in a distant, imagined past environment. Thus although behaviours may appear maladaptive now, they are present because in that past environment, they were advantageous. Against this, Boyd suggests that many apparently maladaptive traits may be explained without reference to a hypothetical past. For if many cultural practices spread through blind imitation, then at least for a time, random variation will result in the spread of some maladaptive behaviours. More importantly, he argues that the strength of social norm enforcement is such that maladaptive social norms may remain stable for extended periods of time. Genetic evolution has made humans into excellent norm enforcers, but it has not specified the content of those norms; it is up to cultural evolution to do that.

This approach sheds new light on debates going back, among other things, to the founding of social anthropology. In the nineteenth century, ethnologists adhering to stadial theories of human progress noted the presence in multiple cultures of practices and ideas which appeared not to cohere with the culture as a whole, but instead seemed to constitute vestigial forms left over from the past. While later anthropology discarded the notion that cultures progressed through a series of fixed stages, diffusionists continued to argue that cultural traits could often, or largely, be explained by reference to a history of transmission from other cultures. Over time, diffusionist explanation came increasingly to be ignored in favour of synchronic and functional analysis—that is, anthropology came to base itself on the methodological assumption that every cultural trait either might have a function, or did have one (Kuper 1983, pp 1–9). According to Tylor’s (1920, p 16) ‘doctrine of survivals’, vestigial cultural traits continued on in new generations simply by ‘force of habit’; but the functionalists rejected this, arguing that traits would only continue, or diffuse, so long as they continued to play a functional role (Stocking 1984, p 152). Thus although diffusion was not deemed unreal, it was relegated at best to a minor explanatory role in comparison to that of social function.

If Boyd is correct, however, diffusion and ‘vestiges’ may be underpinned by more plausible mechanisms than mere force of habit. Individuals and groups may copy each other blindly, or as a result of some other cue, as with prestige-biased transmission. A social norm may at one point have provided a fitness advantage in the context of cultural group selection, but once that context falls away, the norm may live on, stabilised by powerful enforcement mechanisms. Even in the presence of selection, local fitness maxima may result in the preservation of traits which may be globally suboptimal. This theory has the benefit not only of being more plausible than a common sense appeal to force of habit, but, because it is rooted in precise evolutionary models, holds the prospect of generating precise predictions about when and to what extent ‘survivals’ will last, depending on selection pressures (Tehrani 2010).

Moreover, Boyd shows how cultural evolutionary research can already test the functionalist claim that cultural traits survive only so long as they serve a social function. In the context of his argument for blind imitation as opposed to intelligent design in cultural evolution, he points out that if intelligence drives adaptation, then the shadow of history should be short. That is, intelligent individuals designing cultural solutions to problems should pay little heed to the practices of their ancestors, unless it happens that those practices are still optimal. The same argument, of course, can be applied to functionalism: if cultural traits are tightly tied to social function, then the shadow of history should be correspondingly short. But as Boyd argues, evidence from the field of cultural phylogenetics shows that this is not the case. Many cultural traits are strongly determined by history, or diffusion, even in the face of apparently contravening present or local functional demands. The shadow of history is long, but finite. It is now possible, then, to give a more nuanced picture than anthropologists on either side of the nineteenth century debate. Imitation and norm enforcement create path dependencies, but the extent of these dependencies depends closely on selection pressures.

Thus as Radcliffe-Brown (1952, pp 1–14) argued, historical and functional explanation in anthropology are, indeed, complementary. But as in evolutionary analysis more generally, the two are interdependent. Historical explanation requires reference to function and selection pressures in the past, while synchronic functional explanation must always be qualified by path dependency and the presence of mutations and transmission errors. An anthropology that takes cultural evolution seriously is one which systematically combines these approaches; for if cultural evolutionary theory is correct, it is just as fruitless to separate them as it is to analyse the functions of an animal’s organs and the evolutionary history of those organs in isolation from one another.

It thus seems that cultural evolution holds the promise of helping to resolve—or dissolve—old debates about whether anthropology should be functionalist, diffusionist, evolutionist, or none of these. If that is the case, then the same may apply similarly to other social sciences, too. Cultural evolution requires no rigid adherence to either functional or historical explanation, materialism or idealism, and so on; instead it offers a toolkit for the study of how a variety of forces may shape any given social phenomenon. Likewise, cultural evolution entails no metaphysical commitment to social wholes, atomistic rational individuals, or similar exotic beasts; instead, it simply promises a social science in which what happens inside brains, and what happens between people, form two inseparable parts of a dynamic, but metaphysically unmysterious causal story (Sperber 1985).

## Extending the Synthesis: Radically Encultured Brains

But while the cultural evolutionists are keen to point out that, contrary to the tendency of evolutionary psychologists to treat human minds as ‘hard-wired’ computers, much human behaviour and concepts are malleable and subject to a much faster form of evolution, it is commonly assumed that for cultural evolution to take place, certain innate mental capacities must be present, such as the ability to imitate, or elements of ‘theory of mind’. Thus with the evolutionary psychologists, many assume that these are mental modules, produced by genetic evolution in some ancient adaptive environment, after which cultural evolution became possible, and built on the foundation of these modules, but did not replace them.

Not so Heyes (2018). On the basis not of any theoretical or ideological opposition, but on reams of empirical evidence collected from recent research in cognitive psychology, Heyes argues that cultural evolution goes much deeper: we are born not with a set of pre-programmed, computer-like modules, but with a set of behavioural tendencies, subtly different from our primate cousins, which, coupled with copious amounts of information from our environment, enables us to develop such mental modules as those required by theory of mind, language, and even motor skills. But that is not to say that these modules do not evolve, for the lesson of cultural evolution is that the very environmental scaffolding which gives rise to them may itself be the carrier of information that is subject to evolutionary forces. Our sophisticated cognitive capacities look like they have evolved because they have done, over many thousands of years, but not by genes alone. We are not blank slates—we do begin life with a ‘starter kit’ which prepares us to develop in interaction with our environment—but neither is what is written on the slate an arbitrary scribble, created anew with each generation. Instead, cultural evolution goes ‘all the way down’.

Heyes acknowledges an affinity to a number of other recent constructivist positions in psychology. Among these, Tomasello’s (2014) ‘shared intentionality hypothesis’ has been particularly influential for scholars of cultural evolution. Tomasello argues that cultural evolution is made possible by a package of genetic adaptations which endow children with the ability to engage in a sharing of intentions, perceptions, and other mental objects. Heyes contrasts her theory with that of Tomasello in part by claiming that, while her arguments are based on cognitive science, his are rooted in Vygotskian psychology. More specifically, while Heyes shows how complex cognitive functions can be constructed out of a basic ‘starter kit’ of subpersonal mechanisms, Tomasello assumes that specifically human cognition must begin with shared intentionality, which must therefore have evolved genetically—with Vygotsky, he is ‘resolutely focused on the most complex kinds of thinking’ (Heyes 2014).

This interpretation of Vygotskian psychology is not without textual support. Much of Vygotsky’s work was preoccupied with the claim that language acquisition leads to a reorganisation of cognitive function; and on several occasions, he appears to claim that preverbal children are akin to apes (Vygotsky 1978, p 24, Vygotsky 1986, pp 86–87). Thus on one interpretation, the Vygotskian view is that the acquisition of language is what enables enculturation to get going, and therefore justifies the existence of cultural-historical psychology (Cole 2005). Tomasello’s innovation here lies in his claim—strongly contested by some self-described Vygotskians (Fernyhough 2005, 2008)—that shared intentionality is a prerequisite for the development of language. If that is the case, and if cultural transmission begins only with language acquisition, then the capacity for shared intentionality must precede cultural transmission, and is therefore presumably genetically inherited.

This is not, however, the only interpretation of Vygotskian psychology. Prominent members of the Vygotskian school, such as the neuropsychologist Luria, claimed repeatedly that the influence of culture on cognition began from the moment of birth, notably including the shaping of motor and perceptual functions through interaction with cultural artefacts (Homskaya 2001, p 87; Luria 1966, p 30; Luria 1976, p 9). Indeed, it might be said that the foundational principle of the whole Soviet school of neuropsychology—itself a precursor of neuropsychology in the West (Goldberg and Bougakov 2009)—was the notion that the ontogeny of the human brain is, from beginning to end, necessarily shaped by interaction with the individual’s social environment (Cagigas and Bilder 2009). Vygotsky and Luria’s preoccupation with ‘higher’ mental functions may simply reflect the paucity of evidence available at the time concerning the role of environmental influences in preverbal development. On this view, then, Heyes’ accomplishment is not to undermine the Vygotskian school—at least in its original, Soviet form—but to bring its key insights up to date with evidence from modern cognitive science.

Heyes does this in two principal ways. First, she shows in detail how cognitive accomplishments as basic as motor skills, and as complex as language, appear to arise during development on the basis of a few simple principles, rather than depending on pre-programmed genetic modules. Imitation, often seen as a task so complex it can only be carried out by virtue of an innate, cognitive black box, is instead explicable as a result of ‘associative sequence learning’—a combination of vertical associations between motor sequences and sensory inputs, themselves formed by the same simple associative learning which enables the development of motor skills. Even language, an area on which Heyes demurs, as a non-specialist, from making a definitive pronouncement, appears to be amenable to this form of explanation; Heyes shows how recent neurological, developmental and mathematical evidence provides a strong case against the Chomskyan view that language must necessarily depend on a universal language module of some form. In all these respects, just as Boyd brings old social scientific intuitions into precise relief in the light of contemporary research, Heyes does the same for developmental psychology; we can now do far better than a vague debate about ‘nature’ versus ‘nurture’.

Secondly, Heyes’ theory represents an important advance on earlier intuitions because while denying that cognitive modules are the direct product of genetic evolution, she shows why it looks as if such modules have evolved. For they have indeed done so, but through cultural evolution, rather than genetic. While traditional evolutionary psychology, motivated in part by the assumption that genetic evolution takes place at a glacial pace, has been preoccupied with hypothetical accounts of the evolutionary pressures under which our prehistoric ancestors lived, the cognitive gadgets model instead suggests that evolutionary forces relevant to psychology are far more recent and rapid. As our understanding of the complex interaction between genes and environment grows, the metaphor of genetic transmission as a process of downloading modules wholesale, as in transmission between two computers, becomes less and less persuasive (Sasaki and Kim 2017). If the cognitive gadgets theory is correct, it implies not a divorce of psychology from evolution, but a correction of a deep misunderstanding of how evolution, and ontogeny, actually take place.

Quite apart from the evolutionary argument, it is worth appreciating what the cognitive gadgets theory might tell us about the direction of psychology as a whole. Half a century ago, the cognitive revolution upended the discipline, drawing in part on the metaphor of the mind as computer to motivate attempts to analyse the inner workings of that computer. As fruitful as the metaphor has been, it must perhaps be held partly responsible, too, for notions such as the ‘hard-wiring’ of the brain, or indeed of hard-wired mental modules. These notions have been strained by modern neuroscientific research, which has increasingly shown the brain not only to be highly plastic, but also to be radically shaped by its changing social environment. Of course, Heyes’ theory retains the modular framework beloved of cognitive scientists, and indeed, far from breaking with cognitive science, she builds incrementally upon it. But her approach arguably represents a deeper shift, which may yet prove as significant as the original cognitive revolution—a shift to a psychology inspired not by metaphors of ‘hard-wiring’ and ‘software’, but by the brain itself, in all its dynamism and variety.

## Redrawing the Disciplinary Map of the Human Sciences

The accounts offered by Boyd and Heyes are largely complementary. Boyd’s argument for a form of cultural evolution led by blind imitation and cultural group selection can clearly be extended to include the evolution of cognitive mechanisms themselves. Indeed, Heyes comes down firmly on the side of cultural group selection, arguing that cognitive gadgets which are today universal may have evolved by virtue of advantages they conferred to those groups which exhibited the cultural norms necessary to scaffold them.

But while Boyd is keen to emphasise that the key to human success is blind imitation, rather than intelligence, if Heyes is right, then it may be that much of what we think of as intelligence is itself a product of the same forces. On that account, the question of whether cultural change proceeds by intelligence or by blind imitation may pose a false dichotomy. Indeed, Heyes’ extension of cultural evolution into the psychological domain goes so far as to call into question most of what cultural evolutionists have hitherto assumed to be innate; she suggests even that, contrary to Boyd and others (Kelly and Davis 2018), normativity and moral psychology itself may be a cognitive gadget.

Nevertheless, while Boyd is concerned mainly with explaining cultural variation, Heyes’ theory is explicitly aimed at explaining a package of cognitive capacities which she takes to be universal, but which she postulates became universal through a process of cultural evolution. But the theory leaves open the possibility, too, of systematically investigating variations in cognitive mechanisms of the kind of which anthropologists have long spoken. In recent decades, the fields of cross-cultural psychology, cognitive anthropology, and related disciplines have begun to document often fine-grained psychological differences between populations, ranging from differences in social cognition to more fundamental variations, such as in visual perception (Keith 2019). A promising extension of Heyes’ thinking would be to begin to fit these findings within the broad cultural evolutionary approach of Boyd and others—allowing us in this way not only to explain cross-cultural psychological variation, but to draw more precisely the boundaries between culturally specific cognitive mechanisms and those which have become universal, and which we might therefore think of as constituting something like ‘human nature’, or at least the nature of humans as we currently stand.

A further possible fusion between Heyes’ thinking and that of broader culture-gene coevolutionary theory is in the telling of the story of human origins. Throughout human prehistory, we find take-off points, at which we suddenly seem to see a leap in cognitive complexity. Evolutionary theorists have often thought of these as resulting from genetic changes, which suddenly endowed humans of the time with new capabilities. But Heyes’ theory offers the possibility for a more nuanced view, in which some of these take-off points might have been the result of sudden cultural shifts—new practices or technologies which provided the scaffolding for the development of new cognitive capacities. Cultural group selection, as in Boyd’s account, may then have led to rapid shifts to new equilibria, producing the sorts of take-off points we find in the archaeological record. Bearing in mind the likely interactions between genetic and cultural evolution, this may be reason to look again at putative explanations for the sudden development of such capabilities as speech, figurative art, and ritual.

But beyond the story of human origins, this approach holds the exciting prospect for both psychology and anthropology that future investigation may be based neither on attempting to work out which human characteristics are ‘hard-wired’, and which are not, nor even on simply estimating the contribution made to characteristics by various forms of inheritance, but on the broader evolutionary stability, trajectory and interaction of both psychological mechanisms and the cultural practices which sustain and result from them. While it will remain important to investigate the role of various forms of inheritance in ontogeny, this approach suggests that, as with developments in epigenetics, the lines between phylogeny and ontogeny may soon begin to blur (Colagè and d’Errico 2018).

In the closing passages of Darwin’s *Origin of Species* (1861, pp 420–425), he outlined with uncanny accuracy the implications his theory would have for the biological sciences over the century to come. In particular, where once these fields had existed as disparate, unconnected endeavours, as diverse as butterfly collecting and palaeontology, Darwin foresaw that evolutionary theory would unite them, by placing them under a single explanatory umbrella. Not only did evolution unite fields by relating the study of different species and genera within a larger family tree of life; it united the study of phylogeny and ontogeny, motivating the latter as a quest for solutions to evolutionary problems, and the former as a means to explain the development of the latter.

However, one key link in Darwin’s chain has remained outside the broader umbrella of the biological sciences. That is, of course, humankind itself. This is not for want of trying; numerous attempts have been made since Darwin’s own time to subsume the study of human behaviour into biology. But such attempts, when not badly misconstruing the concept of evolution and natural selection, have consistently been based on the idea that evolutionary explanation applies only to inheritance by reproduction. Thus they either attempt to fit human behaviour into a procrustean bed of questionable universals, or they provide only a highly circumscribed role for evolutionary explanation.

Cultural evolution promises to change this. For the field as a whole is pointing increasingly toward the potential for a synthesis of just the kind Darwin predicted, in at least two ways. Firstly, if the cultural evolution of cognition turns out to be right, this would mean that already questionable disciplinary distinctions between psychology and anthropology—and indeed, between the study of individuals and broader social sciences more generally—may have to be jettisoned. Just as many of the old disciplinary boundaries of biology ceased to make sense in light of the evolutionary synthesis, the cultural evolution of cognition may force us to think of psychology as fundamentally cultural, and culture as fundamentally psychological.

Secondly, bringing cultural variation under the rubric of evolutionary models holds the potential to complete the evolutionary synthesis, by subsuming human behaviour under the same umbrella as the rest of life, without ignoring the very real cultural variation which defines so much of what it is to be human (Muthukrishna and Henrich 2019). Contrary to the arguments of earlier generations of sociobiologists and their opponents, this approach does not require a ‘reduction’ of humanity to something less than human in order to explain our nature. Instead, by recognising that evolutionary models can apply to a wide variety of forms of information transmission—not simply genes alone—cultural evolution shows how the enormous variation that makes us perhaps most distinctive as a species can be understood within the same broad framework with which we have come to view the whole family tree of life. To break down existing barriers not only between psychology and the social sciences, but between the study of humans and of the rest of life, would be to fulfil the ultimate promise of evolutionary theory; but it would also be to demonstrate the profound beauty of a theory which, in the final analysis, is general enough to apply to everything from the most sublime works of human creativity to the humble constructions of an earthworm.
